# miRNA-378 reverses chemoresistance to cisplatin in lung adenocarcinoma cells by targeting secreted clusterin

**DOI:** 10.1038/srep19455

**Published:** 2016-01-19

**Authors:** Xuesong Chen, Ying Jiang, Zheping Huang, Dandan Li, Xiaodi Chen, Mengru Cao, Qingwei Meng, Hui Pang, Lichun Sun, Yanbin Zhao, Li Cai

**Affiliations:** 1 Department of Internal Medical Oncology, Harbin Medical University Cancer Hospital, Harbin, Heilongjiang Province, China; 2 Department of Biomedical and Pharmaceutical Sciences, College of Pharmacy, University of Rhode Island, Kingston, RI, USA; 3 Department of Pediatrics, The Alpert Medical School of Brown University, Women & Infants Hospital of Rhode Island, Providence, RI, 02905.

## Abstract

Cisplatin resistance is a major obstacle in the treatment of NSCLC, and its mechanism has not been fully elucidated. The objectives of the study were to determine the role of miR-378 in the sensitivity of lung adenocarcinoma cells to cisplatin (cDDP) and its working mechanism. With TargetScan and luciferase assay, miR-378 was found to directly target sCLU. miR-378 and sCLU were regulated in A549/cDDP and Anip973/cDDP cells to investigate the effect of miR-378 on the sensitivity and apoptotic effects of cDDP. The effect of miR-378 upregulation on tumor growth was analyzed in a nude mouse xenograft model. The correlation between miR-378 and chemoresistance was tested in patient samples. We found that upregulation of miR-378 in A549/cDDP and Anip973/cDDP cells significantly down-regulated sCLU expression, and sensitized these cells to cDDP. miR-378 overexpression inhibited tumor growth and sCLU expression in a xenograft animal model. Analysis of human lung adenocarcinoma tissues revealed that the cDDP sensitive group expressed higher levels of miR-378 and lower levels of sCLU. miR-378 and sCLU were negatively correlated. To conclude, we identified sCLU as a novel miR-378 target, and we showed that targeting sCLU via miR-378 may help disable the chemoresistance against cisplatin in lung adenocarcinoma cells.

Lung cancer is the leading cause of cancer-related death in both males and females worldwide^1^. Non-small cell lung cancer (NSCLC) is the most common type of lung cancer, accounting for 80–85% of all lung cancer cases^2^. Cisplatin, a DNA-damaging cytotoxic agent, is the first-line therapy in NSCLC treatment, but its efficacy is often impaired by the development of drug resistance^3^. Although lots of studies have been done on the resistance to cisplatin^3^,^4^, the mechanisms involved are not fully understood, so further study is needed.

Clusterin (CLU) is a secreted glycoprotein which is involved in many physiological processes, such as apoptosis, cell cycle regulation, and DNA repair^5^,^6^,^7^. It has two main isoforms: secreted form (sCLU) and nuclear form (nCLU). A previous study in our lab showed that sCLU is associated with resistance to cDDP in NSCLC^8^. Other reports have also demonstrated that sCLU is a key contributor to chemoresistance to anticancer agents^6^. sCLU is expressed in aggressive late stage tumors. Its expression can lead to the development of broad-based resistance to different chemotherapeutic agents. Inhibition of sCLU could improve the effect of chemotherapy on human tumor cells^9^.

MicroRNAs are a class of short, non-coding RNA molecules involved in numerous biological processes, such as cell self-renewal and cancer development^10^. By binding with the 3′ untranslated region (UTR) of target mRNAs, miRNA works as a guide molecule in post-transcriptional gene silencing, leading to degradation of target mRNA, or repression of translation^11^. A growing body of evidence suggests that miRNAs may be involved in the development of chemoresistance, and may also play a role in the modulation of drug resistance-related pathways in cancer cells^12^,^13^,^14^.

In this report, we demonstrated that miR-378 targets sCLU, and explored its possible roles in chemoresistance to cDDP in lung adenocarcinoma cells. With TargetScan software, we found a group of miRNAs (including miR-378) that target CLU. Meanwhile, we found two sets of observations that helped us to focus our research on miR-378. Using the microRNA microarray to examine tumor microRNA expression patterns, Eitan *et al*. found that seven microRNAs had significantly different expressions in tumors from platinum-sensitive vs. platinum-resistant patients^15^. In another article, Lu discovered that 34 miRNAs were differentially expressed between the A549 and A549/cDDP cell line^16^. Both reports demonstrated that miR-378 might be important in the chemoresistance to platinum, but the mechanisms for the effect remain poorly understood. Therefore, we selected miR-378 as the target of our research.

Here we demonstrate that miRNA-378 regulates sCLU-mediated chemosensitivity to cDDP in non-small cell lung cancer cells.

## Results

### miR-378 directly targets sCLU

**Figure 1:**
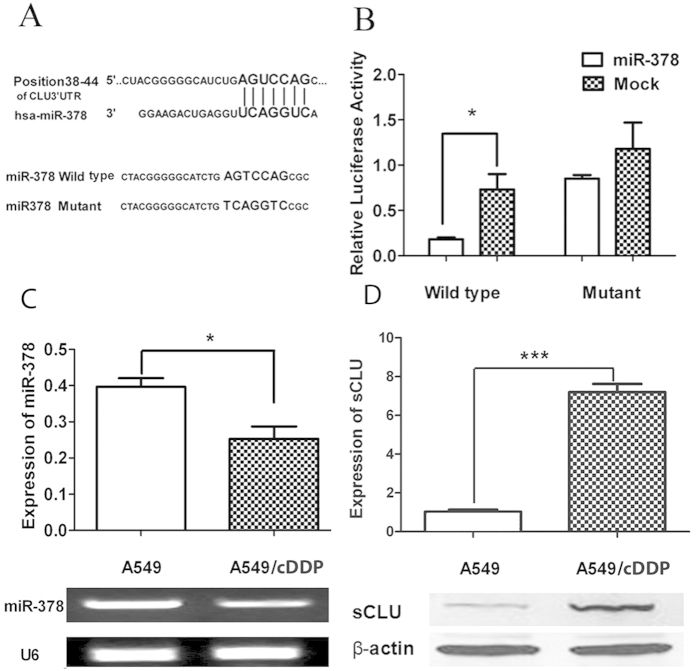
miR-378 directly Targets sCLU. (**A**) The upper panel is the predicted miR-378 binding site with CLU3′UTR. The lower panel is the miR-378 sequence containing the putative sCLU binding site and its mutant sequence. (**B**) Dual luciferase assay was performed with co-transfection of miR-378 mimics and the reporter containing sCLU 3′-UTR wild-type versus mutant. (**C**) RT-PCR results showed that A549 cells had higher expression of miR-378 compared with A549/cDDP cells. The lower panel is the representative picture of DNA electrophoresis after PCR. (**D**) The Western blot analysis showed the protein level of sCLU in A549 cells and A549/cDDP cells. The lower panel showed the representative blots. All results are from three separate experiments. (*P < 0.05, ***P < 0.001. Full-length blots are presented in Supplement.).

As our previous research showed that sCLU is related to the development of chemoresistance to cDDP, We used the bioinformatics tool (TargetScan, www.targetscan.org) to search the miRNAs that may target sCLU, which revealed many candidates (including miR-378). As two sets of observations showed that miR-378 is important in chemoresistance to cDDP^15^,^16^, and as we found that miR-378 was identified as one of the miRNA that target sCLU, we focused our research on miR-378. As shown in Fig. 1A, the sequence of miR-378 was partially complementary to the 3′ untranslated region (UTR) of the sCLU mRNAs (7 nucleotides completely match). To verify that miR-378 targets sCLU, we cloned a fragment of CLU 3′UTR containing the putative miR-378 binding site, or its mutated one, into a luciferase reporter vector, and performed dual luciferase assays in 293T cells. The results showed that up-regulation of miR-378 significantly decreased the relative luciferase activity of wild type but had no effect on the mutant 3′UTR of sCLU (Fig. 1B). Next, we compared the expression of miR-378 and sCLU in the cisplatin-resistant A549 cell line (A549/cDDP) with its parental A549 cell line. The A549/cDDP cell line had a lower level of miR-378 and a higher level of sCLU compared with the A549 cell line (Fig. 1C,D). In addition, our previous research showed that Anip973/cDDP had a higher level of sCLU^8^, and here we showed that Anip973/cDDP had a lower level of miR-378 compared with the Anip973 (supplementary Figure 1A).Taken together, these results suggest that miR-378 suppresses sCLU gene expression through the 3′-UTR binding and silencing of sCLU mRNA. The A549/cDDP and Anip973/cDDP cell lines were used in later experiments due to its higher expression of sCLU.

### miR-378 increases sensitivity of A549/cDDP to cDDP and apoptosis through sCLU

**Figure 2:**
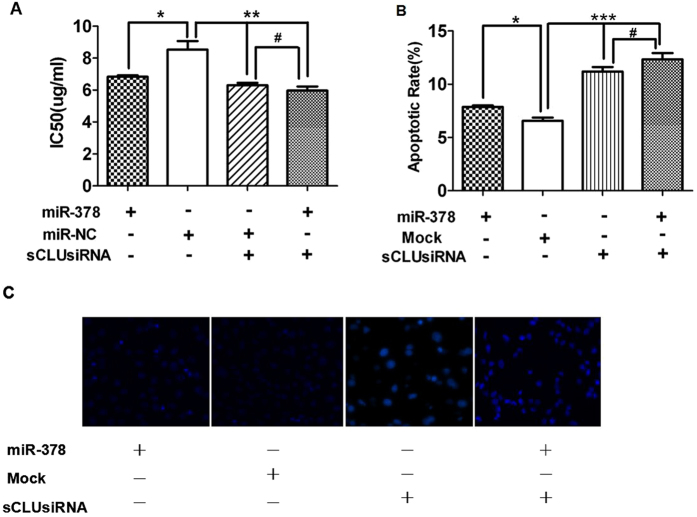
miR-378 and sCLU affects A549/cDDP cell’s sensitivity to cDDP and apoptosis in cells. (**A**) MTT assay results showed that both miR-378 overexpression or sCLU knockdown in A549/cDDP significantly decreased the IC_50_; after sCLU knockdown, miR-378 overexpression had no effect. (**B**,**C**) Hoechst staining assay showed that the apoptosis rate significantly increased in miR-378 overexpression cells compared with vector controls; after sCLU knockdown, miR-378 overexpression had no effect on apoptosis. (*P < 0.05, **P < 0.01, ***P < 0.001, ^#^P > 0.05).

To test miR-378’s effect on A549/cDDP’s sensitivity to cDDP, MTT assay was performed. The results showed that overexpression of miR-378, or knockdown of sCLU, both sensitized the cells to cDDP (Fig. 2A). Similar results also could be seen in Anip973/cDDP, another cell line with high CLU expression^8^. After sCLU knockdown, miR-378 transfection had no effect on the sensitivity of the cells (Supplementary Figure 1B). In addition, overexpression of miR-378 in A549 cells or Anip973 (both with low CLU expression^8^) also had no effect on the sensitivity of the cells. miR-378 transfection sensitized these cells to cDDP only after sCLU overexpression (supplementary Figure 2).

We went further to test miR-378’s effect on apoptosis. Hoechst staining assay showed that either downregulation of sCLU or overexpression of miR-378 increased cell apoptosis in A549/cDDP cells and Anip973/cDDP. On the contrary, after sCLU knockdown, transfection of miR-378 did not change the apoptotic rate (Fig. 2B,C, and supplementary Figure 3). These results showed that miR-378’s effect on the sensitivity of the cells to cDDP or apoptosis depended on the presence of higher levels of sCLU, so it strongly indicated that miR-378 affected sensitivity of the cell and apoptosis through sCLU.

### sCLU is involved in miR-378-induced cisplatin resistance *in vitro*

**Figure 3:**
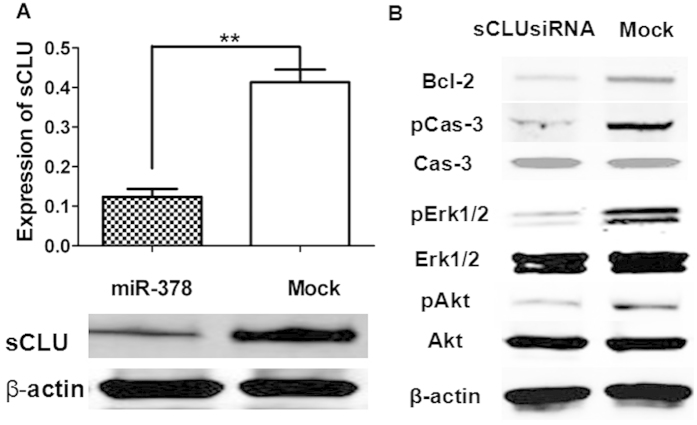
miR-378 targets sCLU and regulated its cell signal. (**A**) Western blot showed upregulation of miR-378 led to a significant decrease of sCLU protein in A549/cDDP cells. Results are from three separate experiments. **P < 0.01. (**B**) Western blot showed that down-regulation of sCLU led to a significant decrease of pERK1/2, pAKT, Bcl-2, and caspase-3 in A549/cDDP cells. Full-length blots are presented in Supplement.

To test whether sCLU is a target of miR-378, Western blot was used to check the effect of miR-378 transfection on sCLU in the A549/cDDP cell line. The result showed that upregulation of miR-378 led to a significant decrease of sCLU protein in A549/cDDP cells (Fig. 3A). The Western blot results also demonstrated that downregulation of sCLU led to a significant decrease of pERK1/2, pAKT, Bcl-2, and pCaspase-3 signal in A549/cDDP cells (Fig. 3B).

**Figure 4:**
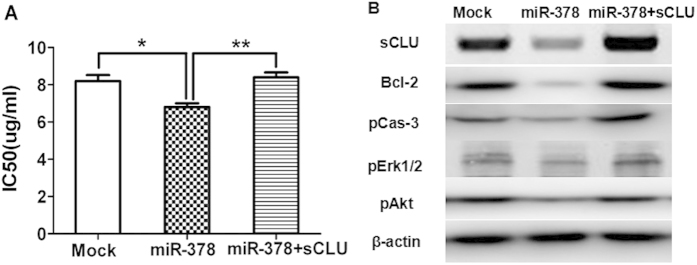
sCLU is involved in miR-378-induced cisplatin resistance. (**A**) A549/DDP cells were transfected with Mock, miR-378 mimics or sCLU plasmid lacking 3′UTR along with miR-378, MTT assays were used to measure cisplatin sensitivity (*P < 0.05 **P < 0.01). (**B**) sCLU, pERK1/2, pAKT, Bcl-2, and caspase-3 protein levels were detected by Western blot analysis.

To further examine whether sCLU is involved in miR-378 induced chemoresistance, we performed gain-of-function and loss-of-function analysis. We transfected A549/cDDP cells with the miR-378 mimics(miR-378), and performed MTT assay. The results showed that upregulation of miR-378 led to a significantly increased sensitivity to cDDP in A549/cDDP cells (Fig. 3B).To test whether the above-observed phenotype was indeed due to the suppression of sCLU, a rescue experiment was performed. We transfected A549/cDDP cell with an expression construct that encoded sCLU coding sequence but lacked the 3′UTR. Forced expression of sCLU eliminated most of the gains in the sensitivity to cDDP in miR-378-overexpressing cells (Fig. 4A), and reversed the decrease of Bcl-2, pCas-3, pErk1/2 and pAkt (Fig. 4B). Collectively, these data strongly suggest that miR-378 regulate chemo-resistance to cDDP by targeting sCLU.

### miR-378 inhibited tumor growth and sCLU expression in a nude mouse tumor xenograft model

**Figure 5:**
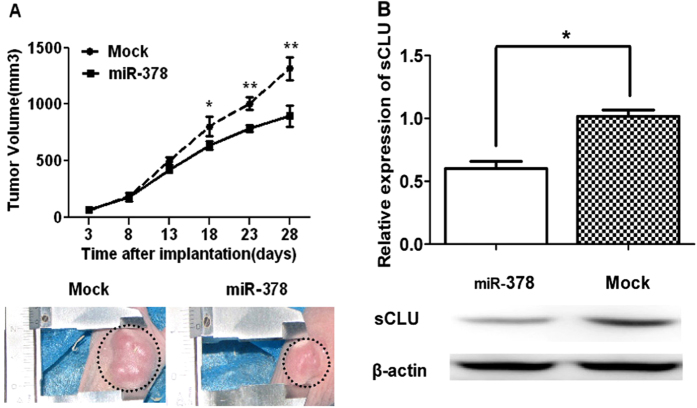
miR-378 inhibited tumor growth and sCLU expression in Nude mouse tumor xenograft model. (**A**) Analysis of the tumor growth curves showed miR-378 significantly inhibited tumor growth 18 days after cell injection (P < 0.05). The upper panel showed the growth curves, and the lower panel showed the representative picture of tumors with miR-378 overexpression (right) and Mock (left); (**B**) Western blot showed miR-378 inhibited sCLU expression. The upper panel showed the results of three separate experiments, and the lower panel showed the representative blot. (*P < 0.05. Full-length blots are presented in Supplement).

To investigate how miR-378 overexpression affected sCLU expression and tumor growth, agomir-378 cells and cells with agomir-NC(Mock) were injected into the flank of nude mice. The tumor growth curve analysis showed that miR-378 significantly inhibited the tumor growth in 18 days after the injection of cells (p < 0.05) (Fig. 5A). Western blot demonstrated that sCLU was significantly down-regulated in the A549/cDDP-miR-378 cells (P < 0.05) (Fig. 5B).

### miR-378 and sCLU correlated with sensitivity to cDDP in lung adenocarcinoma tissues

**Figure 6:**
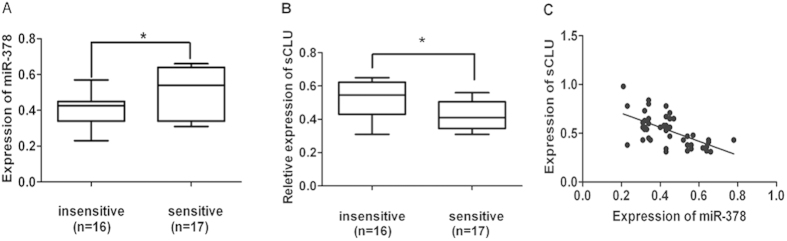
miR-378 and sCLU are correlated with sensitivity to cDDP in lung adenocarcinoma tissues. (**A**) RT-PCR results showed that miR-378 expression levels were significantly lower in the insensitive group than in the sensitive group, *P < 0.05; (**B**) The Western blot analysis showed that sCLU levels were significantly higher in the insensitive group compared with the sensitive group (*P < 0.05). C. Correlation analysis shows that miR-378 negatively correlates with the sCLU mRNA expression level, r = −0.538. (*P < 0.05).

To investigate the association between miR-378 and sCLU expression, 33 tumor tissue samples were obtained from patients with advanced lung adenocarcinoma. The characteristics of the patients were detailed in table 1 (see supplement). Based on the patients’ response to chemotherapy, they were divided into “sensitive” and “insensitive” groups, and the miR-378 levels and sCLU expression levels of each sample were analyzed. We found that miR-378 expression level was significantly lower in the “insensitive” group tissues (0.40 ± 0.02) compared to the “sensitive” group (0.51 ± 0.03) ( P < 0.05) (Fig. 6A). On the contrary, sCLU levels were significantly higher in the “insensitive” group tissues (0.51 ± 0.03vs 0.42 ± 0.02) (P < 0.05) (Fig. 6B). Correlation analysis showed that miR-378 was negatively correlated with sCLU expression level (r = −0.538). (Fig. 6C)

## Discussion

Resistance to cisplatin-based chemotherapy is one of the major obstacles in the treatment of NSCLC^17^. Evidence demonstrates that dysregulation of specific miRNAs causes the development of chemoresistance in different cancers^18^,^19^. In this study, we identified sCLU as a novel target of miR-378; we demonstrated that miR-378 overexpression could decrease sCLU, enhance cell apoptosis, and sensitize lung adenocarcinoma to cDDP both *in vitro* and *in vivo*. To our knowledge, our study is the first to demonstrate the association of miR-378 with the development of cDDP chemoresistance in human lung adenocarcinoma.

Drug resistance can develop at many levels, including increase of drug efflux, alterations in drug target, cell cycle regulation, DNA repair, and evasion of apoptosis. It has been shown previously that selective regulation of microRNA activity can improve responsiveness to chemotherapy^20^. miR-378 expression is found in a number of cancer cell lines^21^,^22^, and is related to the expression of vascular endothelial growth factor^23^,^24^. Also, miR-378 is shown to be important in chemoresistance to cDDP, but no detailed mechanism is reported^15^,^16^. We found that miR-378 is partially complementary to the 3′ untranslated region (UTR) of the CLU mRNAs using Bioinformatics (TargetScan) (Fig. 1A), and miR-378 can affect the luciferase activity due to canonical binding to sCLU 3′-UTR (Fig. 1B). This data is corroborated by the expression of miR-378 and sCLU in A549 vs A549/cDDP (Fig. 1C,D) and Anip973 vs Anip973/cDDP^8^ (supplementary Figure 1A). Thus, we clearly established an inverse relationship between miR-378 and sCLU. Furthermore, overexpression of miR-378 can reduce the sCLU level (Fig. 3A), sensitize A549/cDDP and Anip973/cDDP cells to cDDP (Fig. 2A, supplement Figures 1B and 4A), and inhibit the tumor growth in nude mice model (Fig. 5A). Moreover, our data are also corroborated by the observations in human tumor tissues obtained from patients who showed sensitivity or insensitivity to cDDP, and we found an inverse correlation between miR-378 and sCLU expression levels in tumor tissues samples.

Interestingly, previous studies have indicated that miR-378 transfection enhanced cell survival, tumor growth, and angiogenesis in NSCLC cells, but target genes were not identified^23^,^24^. Also, there are some inconsistent results. Wang *et al*. found that miR-378 inhibited cell growth and enhanced L-OHP-induced apoptosis in human colorectal cancer by targeting CDC40^25^. Fei *et al*. reported that miR-378 suppress HBV-related hepatocellular carcinoma tumor growth by directly targeting the insulin-like growth factor 1 receptor^26^. A single miRNA has the potential to disrupt multiple pathways involved in regulating cancer cell survival or drug response. With this in mind, the effect of miR-378 was determined by the function of target genes in the current study.

Clusterin (CLU), in its cytoplasmic secretory form (sCLU), has been demonstrated to mediate chemoresistance to numerous unrelated anticancer agents, and its presence has been observed in a variety of solid tumors and lymphomas^27^,^28^. sCLU can also mediate the development of resistance to targeted death-inducing molecules, tumor necrosis factor, Fas, TRAIL, or histone deacetylase inhibitors^4^,^5^,^6^. sCLU is expressed in around 40% of human NSCLC^7^,^29^, and down-regulation of sCLU by sequence-specific antisense oligonucleotides or siRNA can enhance paclitaxel chemosensitivity in human lung cancer cells^6^. nCLU is generally associated with cell death^30^,^31^,^32^,^33^, but it does not exist in non-small cell lung cancer^7^. Downregulation of sCLU sensitized the DDP-based chemotherapy in human osteosarcoma cell and in human non-small cell lung cancer xenografts in immunodeficient mice by inactivating downstream cell signal pAKT and pERK1/2^34^,^35^. In contrast, overexpression of sCLU in MCF-7/sClu cells promoted TNF-alpha-mediated NF-kappaB activity and Bcl-2 overexpression^36^. Here our research further confirms that sCLU plays an important role on sensitivity to cDDP in human lung adenocarcinoma cells by downregulating Bcl-2, pCas-3, pErk1/2 and pAkt.

**Figure 7:**
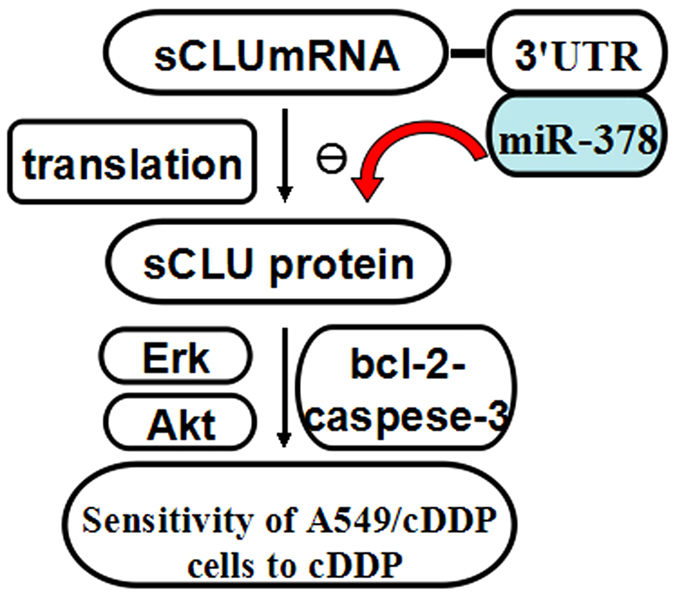
A mechanistic model of regulation of lung adenocarcinoma’s sensitivity to cDDP via miR-378 and sCLU. Upregulation of miR-378 in A549/cDDP cells significantly down-regulates sCLU expression, and enhances the sensitivity of A549/cDDP cells to cDDP.

In conclusion, we report altered expression of miR-378 in human lung adenocarcinoma cell lines with varying sensitivities to cDDP, and have shown that miR-378 can restore cDDP chemosensitivity in the human lung adenocarcinoma cells by targeting sCLU and downregulating Bcl-2, pCas-3, pErk1/2 and pAkt (Fig. 7). Therefore, targeting this miR-378-sCLU interaction may be a potential strategy for reversing cDDP chemoresistance in human lung adenocarcinoma.

## Methods

### Cell culture

All human lung adenocarcinoma cell lines (A549, Anip973, A549/cDDP, Anip973/cDDP) were purchased from the Cancer Research Institute of Heilongjiang Province [Harbin, China, (STR profiling)] and passaged in our laboratory for fewer than six months after resuscitation. The cells were grown in RPMI-1640 medium with 10% fetal calf serum under atmospheric conditions of 5% CO2 with humidity at 37 °C.

### MTT assay

MTT assay was performed as described previously^37^. Briefly, the stably transfected cells were seeded into 96-well plates and incubated overnight. Freshly prepared cDDP was added. After 48 h, MTT solution (5 mg/mL) was added (20 μl/well), and cells were incubated for 4 more hours. The culture supernatant was removed and DMSO was added (100 μl/well). Cells were incubated in a shaker at 37 °C for 10 min until the crystals were completely dissolved. A microplate reader was used to measure the absorbance at 490 nm. The concentration at which the drug produced 50% inhibition of growth (IC50) was estimated by the relative survival curve.

### miRNA and transfection

miR-378 mimics, sCLU siRNA, sCLU(plasmid) or their controls were purchased from Sigma. Transfection was performed with Lipofectamine 2000 (Invitrogen, Mountain View, CA, USA) following the manufacturer’s protocol. 48 h after transfection, cells were collected for RNA isolation or Western blot.

### Hoechst staining assay

Cells were cultured on six-well plates and treated with cDDP for 12 h. Then, Hoechst 33342 (Sigma, USA) was added to the medium. The changes in nuclear morphology were analyzed with fluorescence microscopy. The percentages of Hoechst-positive nuclei per optical field were counted.

### RNA extraction and RT-PCR assay

Total RNA was isolated with TRIzol Reagent (Invitrogen, Gaithersburg, MD). RT-PCR was performed using TaKaRa one-step RNA PCR kit (TaKaRa Bio Inc, Japan). The expression level of miR-378 was detected with gene-specific primers as described^38^. U6 was validated as an internal control by comparing its expression levels in each of the specimens.

### Western blot

The proteins from cell lysates were separated on 12% SDS–polyacrylamide gels and blotted onto PVDF membranes. All gels have been run under the same experimental conditions. Membranes were then probed with an antibody. Antibodies against sCLU, pCaspase-3, Caspase and Bcl-2 were purchased from Santa Cruz Biotechnology (Santa Cruz, CA). Antibodies against pERK1/2, ERK1/2, pAKT, AKT were purchased from Abcam (Cambridge, MA). We used BCIP/NBT color development system to detect the bands. Images were photographed using the Bio-Rad gel imaging system (Bio-Rad, CA).

### Dual Luciferase Activity Assay

To generate luciferase report constructs, the 3′ UTR of sCLU was amplified by PCR and cloned downstream of the luciferase-coding sequence in the psicheck-2 vector between Xho I and Not I restriction site. Mutations were introduced into the miRNA-binding sites by using the QuickChange Mutagenesis Kit (Stratagene). HEK293T cells were seeded in a 24-well plate and transfected with 20 μM of either miR-378 mimics or miR-NC vectors, and 50 ng of psicheck-2 vectors. Cells were harvested for luciferase activity assays 48 hours after transfection. A luciferase assay kit (dual-luciferase assay kit, E2920, Promega) was used according to the manufacturer’s protocol.

### Animal experiment

A549/cDDP cells (5 × 10^6^ in 0.2 mL of HBSS) were suspended in serum-free RPMI/Matrigel mixture and were injected into the flanks of BALB/c nude mice under isoflurane inhalation (Nu/Nu, female, 4–6 weeks old, n = 5/group), which were maintained under pathogen-free conditions. One day after tumor cell implantation, mice were treated with cDDP (3.0 mg/kg body weight; i.p., thrice per week), and health of mice was checked every day. After 8 days, Agomir-miR-378 or agomir-NC (RiboBio Co., Ltd, Guangzhou, China) was directly injected into the implanted tumor at the dose of 1 nmol per mouse every 4 days for seven times. Tumor volume was monitored for 4 weeks and measured once weekly. The tumor volume formed was calculated by the following formula: Volume = (Length × width^2^) × 0.5. Mice were euthanized by cervical dislocation. Tumors were harvested; half of each tumor was frozen in liquid nitrogen and stored at −80 °C; the other half was fixed in 4% paraformaldehyde and stored in 70% ethanol. The study was approved by the Ethics Committee of Harbin Medical University, and carried out in accordance with approved guidelines.

### Patients and tissue samples

33 cases of tissue samples were obtained from patients who were diagnosed with advanced lung adenocarcinoma and had received chemotherapy in the Third Affiliated Hospital of Harbin Medical University, Heilongjiang Province (China). Patients met all of the 4 following requirements: 1) histological diagnosed with primary lung adenocarcinoma, 2) had at least one measurable lesion, 3) clinically classified as stage IIIB-IV, 4) being treated with cisplatin. Samples were divided into “sensitive” (complete response or partial response) and “insensitive” (stable disease or progressive disease) groups after 2 cycles of cisplatin-based chemotherapy. The study was approved by the Ethics Committee of Harbin Medical University, and written informed consents were obtained from the participants. The study was carried out in accordance with the approved guidelines.

## Statistical Analysis

Each experiment was repeated at least 3 times. Numerical data were presented as mean ± SD. The difference between means was analyzed with Student’s t-test. The correlation was assessed by the Pearson coefficient. All statistical analyzes were performed using SPSS11.0 software. Differences were considered significant when P < 0.05.

## Additional Information

**How to cite this article**: Chen, X. *et al*. miRNA-378 reverses chemoresistance to cisplatin in lung adenocarcinoma cells by targeting secreted clusterin. *Sci. Rep.* **6**, 19455; doi: 10.1038/srep19455 (2016).

## Supplementary Material

Supplementary Information

## References

[b1] Jemal A. . Cancer statistics, 2009. CA Cancer J Clin. 59, 225–249 (2009). 19474385 10.3322/caac.20006

[b2] Reck M., Heigener D. F., Mok T., Soria J. C. & Rabe K. F. Management of non-small-cell lung cancer: recent developments. Lancet. 382, 709–719 (2013). 23972814 10.1016/S0140-6736(13)61502-0

[b3] Davidoff A. J., Tang M., Seal B. & Edelman M. J. Chemotherapy and survival benefit in elderly patients with advanced non-small-cell lung cancer. J Clin Oncol. 28, 2191–2197 (2010). 20351329 10.1200/JCO.2009.25.4052

[b4] Lee C. H. . Suppression of clusterin expression enhanced cisplatin-induced cytotoxicity on renal cell carcinoma cells. Urology. 60, 516–520 (2002). 12350509 10.1016/s0090-4295(02)01806-x

[b5] Hoeller C. . Clusterin regulates drug-resistance in melanoma cells. J Invest Dermatol. 124, 1300–1307 (2005). 15955107 10.1111/j.0022-202X.2005.23720.x

[b6] Djeu J. Y. & Wei S. Clusterin and chemoresistance. Adv Cancer Res. 105, 77–92 (2009). 19879424 10.1016/S0065-230X(09)05005-2 PMC3889866

[b7] Albert J. M. . Cytoplasmic clusterin expression is associated with longer survival in patients with resected non-small cell lung cancer. Cancer Epidemiol Biomarkers Prev. 16, 1845–1851 (2007). 17855704 10.1158/1055-9965.EPI-07-0146

[b8] Chen X. . Expression and Significance of Clusterin in Anip973/CDDP Cell Lines. Chin J lung cancer. 12, 1074–1078 (2009). 10.3779/j.issn.1009-3419.2009.10.04 20723345

[b9] Djeu J. Y. & Wei S. Clusterin and chemoresistance. Adv Cancer Res. 105, 77–92 (2009). 19879424 10.1016/S0065-230X(09)05005-2 PMC3889866

[b10] Di Leva G., Garofalo M. & Croce C. M. MicroRNAs in Cancer. Ann Rev Pathol. 9, 287–314 (2014). 24079833 10.1146/annurev-pathol-012513-104715 PMC4009396

[b11] Lujambio A. & Lowe S. W. The microcosmos of cancer. Nature. 482, 347–355 (2012). 22337054 10.1038/nature10888 PMC3509753

[b12] Du L. . miR-337-3p and its targets STAT3 and RAP1A modulate taxane sensitivity in non-small cell lung cancers. PLoS One. 7, e39167 (2012). 22723956 10.1371/journal.pone.0039167 PMC3377607

[b13] Feng B., Wang R., Song H. Z. & Chen L. B. MicroRNA-200b reverses chemoresistance of docetaxel-resistant human lung adenocarcinoma cells by targeting E2F3. Cancer. 118, 3365–3376 (2012). 22139708 10.1002/cncr.26560

[b14] Tan S., Wu Y., Zhang C. Y. & Li J. Potential MicroRNA Targets for Cancer Chemotherapy. Curr Med Chem. 20, 3574–3581 (2013). 23834181 10.2174/0929867311320290003

[b15] Eitan R. . Tumor microRNA expression patterns associated with resistance to platinum based chemotherapy and survival in ovarian cancer patients. Gynecol Oncol. 114, 253–259 (2009). 19446316 10.1016/j.ygyno.2009.04.024

[b16] Lu X., Sun J., Gao W., Xu X. T. & Shu Y. Q. Analysis of microRNAs in drug-resistant NSCLC cell line A549/DDP. Chin J Cancer Prev Treat. 179, 659–662 (2010).

[b17] Kim E. S. . Tissue platinum concentration and tumor response in non-small-cell lung cancer. J Clin Oncol. 30, 3345–3352 (2012). 22891266 10.1200/JCO.2011.40.8120 PMC3438232

[b18] Bian H. B., Pan X., Yang J. S., Wang Z. X. & De W. Upregulation of microRNA-451 increases cisplatin sensitivity of non-small cell lung cancer cell line (A549). J Exp Clin Cancer Res. 30, 20 (2011). 21329503 10.1186/1756-9966-30-20 PMC3051912

[b19] Du L. & Pertsemlidis A. microRNA regulation of cell viability and drug sensitivity in lung cancer. Expert Opin Biol Ther. 12, 1221–1239 (2012). 22731874 10.1517/14712598.2012.697149

[b20] Garofalo M. & Croce C. M. MicroRNAs as therapeutic targets in chemoresistance. Drug Resist Update. 16, 47–59 (2013). 10.1016/j.drup.2013.05.001 PMC3858390 23757365

[b21] Deng Z. . The intermediate filament vimentin mediates microRNA miR-378 function in cellular self-renewal by regulating the expression of the Sox2 transcription factor. J Biol Chem. 288, 319–331 (2013). 23135265 10.1074/jbc.M112.418830 PMC3537029

[b22] Deng H. . MicroRNA-195 and microRNA-378 mediate tumor growth suppression by epigenetic regulation in gastric cancer. Gene. 518, 351–359 (2013). 23333942 10.1016/j.gene.2012.12.103

[b23] Chen L. T. . MicroRNA-378 is associated with non-small cell lung cancer brain metastasis by promoting cell migration, invasion and tumor angiogenesis. Med Oncol. 29, 1673–1680 (2012). 22052152 10.1007/s12032-011-0083-x

[b24] Skrzypek K. . Interplay Between Heme Oxygenase-1 and miR-378 Affects Non-Small Cell Lung Carcinoma Growth, Vascularization, and Metastasis. Antioxid Redox Signal. 19, 644–660 (2013). 23617628 10.1089/ars.2013.5184 PMC3740397

[b25] Wang K. Y. . MicroRNA-378 inhibits cell growth and enhances L-OHP-induced apoptosis in human colorectal cancer. IUBMB Life. 66, 645–654 (2014). 25328987 10.1002/iub.1317

[b26] Li L.H., Gao Q., Wang X.Y. & Guo Z.J. miR-378 suppresses HBV-related hepatocellular carcinoma tumor growth by directly targeting the insulin-like growth factor1receptor. ZhonghuaGanZangBingZaZhi. 21, 609–613 (2013). 10.3760/cma.j.issn.1007-3418.2013.08.011 24119742

[b27] Kapoor S. Clusterin inhibition to enhance tumor chemosensitivity in systemic tumors. Cancer Chemother Pharmacol . 71, 1101 (2013). 23385781 10.1007/s00280-013-2072-6

[b28] Muramaki M. . Chemosensitization of gemcitabine-resistant human bladder cancer cell line both *in vitro* and *in vivo* using antisense oligonucleotide targeting the anti-apoptotic gene, clusterin. BJU Int . 103, 384–390 (2009). 19007378 10.1111/j.1464-410X.2008.08098.x

[b29] Cheng C. Y. . Regulation of chemosensitivity and migration by clusterin in non-small cell lung cancer cells. Cancer Chemother Pharmacol. 69, 145–154 (2012). 21630085 10.1007/s00280-011-1682-0

[b30] Caccamo A. E., Desenzani S., Belloni L., Borghetti A. F. & Bettuzzi S. Nuclear clusterin accumulation during heat shock response: implications for cell survival and thermo-tolerance induction in immortalized and prostate cancer cells. J Cell Physiol. 207, 208–219 (2006). 16331665 10.1002/jcp.20561

[b31] Leskov K. S., Klokov D. Y., Li J., Kinsella T. J. & Boothman D. A. Synthesis and functional analyzes of nuclear clusterin, a cell death protein. J Biol Chem . 278, 11590–11600 (2003). 12551933 10.1074/jbc.M209233200

[b32] Moretti R. M. . Molecular mechanisms of the antimetastatic activity of nuclear clusterin in prostate cancer cells. Int J Oncol. 39, 225–234 (2011). 21573492 10.3892/ijo.2011.1030

[b33] Kim N. . Human nuclear clusterin mediates apoptosis by interacting with Bcl-XL through C-terminal coiled-coil domain. J Cell Physiol . 227, 1157–1167 (2012). 21567405 10.1002/jcp.22836

[b34] Huang H., Wang L., Li M., Wang X. & Zhang L. Secreted clusterin (sCLU) regulates cell proliferation and chemosensitivity to cisplatin by modulating ERK1/2 signals in human osteosarcoma cells. World J Surg Oncol. 12, 255 (2014). 25106434 10.1186/1477-7819-12-255 PMC4249734

[b35] Zhang B., Liu Z. M., Hao F. G. & Wang M. siRNA-directed clusterin silencing promotes cisplatin antitumor activity in human non-small cell lung cancer xenografts in immunodeficient mice. Eur Rev Med Pharmacol Sci. 18, 1595–1601 (2014). 24943969

[b36] Wang Y., Wang X., Zhao H., Liang B. & Du Q. Clusterin confers resistance to TNF-alpha-induced apoptosis in breast cancer cells through NF-kappaB activation and Bcl-2 overexpression. J Chemother. 24, 348–57 (2012). 23174100 10.1179/1973947812Y.0000000049

[b37] Chen X. .Tea polyphenols induced apoptosis of breast cancer cells by suppressing the expression of Survivin. Sci Rep. 4, 4416 (2014). 24646833 10.1038/srep04416 PMC3960584

[b38] Lee D. Y., Deng Z., Wang C. H. & Yang B. B. MicroRNA-378 promotes cell survival, tumor growth, and angiogenesis by targeting SuFuand Fus-1 expression. Proc Natl Acad Sci USA. 104, 20350–20355 (2007). 18077375 10.1073/pnas.0706901104 PMC2154434

