# Supplementary material for: miRNA-378 reverses chemoresistance to cisplatin in lung adenocarcinoma cells by targeting secreted clusterin
Source: Sci Rep. 2016 Jan 19;6:19455. doi: 10.1038/srep19455 (PMC4725983; doi:10.1038/srep19455)
Supplement: Supplementary Information [file srep19455-s1.doc]

**miRNA-378 reverses chemoresistance to cisplatin in lung adenocarcinoma cells by targeting secreted clusterin**

Xuesong Chen^1^*, YingJiang^1*^, Zheping Huang^2^*, Dandan Li^1^,Xiaodi Chen^3^, Mengru Cao^1^, Qingwei Meng^1^, Hui Pang^1^, Lichun Sun^1^, Yanbin Zhao^1¶^ , Li Cai^1¶^

Table1. The characteristic and clinicopathological parameters of the patients.

|  | | |  | Drug response | | | | | *P* | |
| --- | --- | --- | --- | --- | --- | --- | --- | --- | --- | --- |
| Variables | No. of Patients | | | | sensitive | | insensitive | | |  |
| Smoking | | |  | | |  | |  | 0.071 | |
| Never | | | 22 | | | 8 | | 14 |  | |
| Ever | | | 11 | | | 8 | | 3 |  | |
| Gender | | |  | | |  | |  | 0.481 | |
| Male | | | 21 | | | 9 | | 12 |  | |
| Female | | | 12 | | | 7 | | 5 |  | |
| Age (years) | | | | | |  | |  | 0.398 | |
| < 60 | | 27 | | | | 12 | | 15 |  | |
| ≥ 60 | | 6 | | | | 4 | | 2 |  | |
| Differentiation | | | | | |  | |  | 0.732 | |
| Well/moderate | | 16 | | | | 7 | | 9 |  | |
| Poor | | 17 | | | | 9 | | 8 |  | |
| AJCC stage | | | | | |  | |  | 0.556 | |
| IIIB | | | 13 | | | 6 | | 7 |  | |
| IV | | | 20 | | | 10 | | 10 |  | |

**
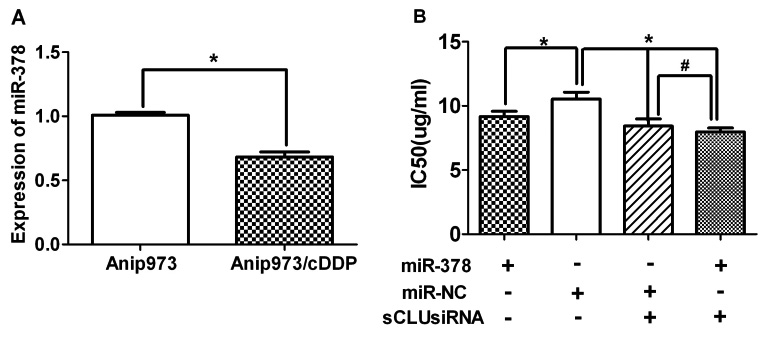
**

**Supplementary Figure 1. Expression of miR-378 and the effect of its overexpression on Anip973/cDDP’s sensitivity to cDDP**

A.RT-PCR results showed that Anip973 cells had higher expression of miR-378 compared with Anip/cDDP cells. (*P<0.05)

B.MTT assay results showed that overexpression of miR-378 or knockdown of sCLU both significantly decreased the IC_50_ compared with their controls in Anip973/cDDP cells; miR-378 overexpression had no effect on IC50 after sCLU knockdown. (*P<0.05, ^#^P>0.05).


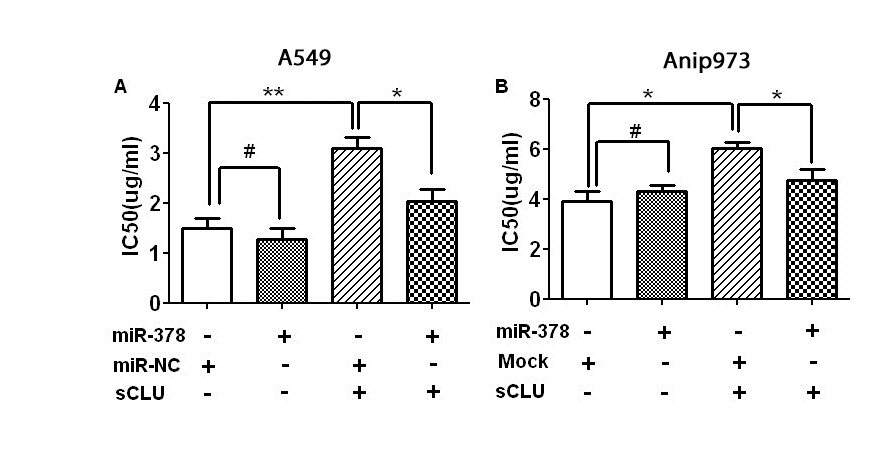


**Supplementary Figure 2. miR-378 overexpression had no effect on A549 or Anip973 cells’ sensitivity to cDDP.**

MTT assay results demonstrated that upregulation of miR-378 had no effect on A549 and Anip973 cell’s sensitivity to cDDP. It affected these cells’ sensitivity to cDDP only after sCLU overexpression. (*P<0.05, **P<0.01, ^#^P>0.05).


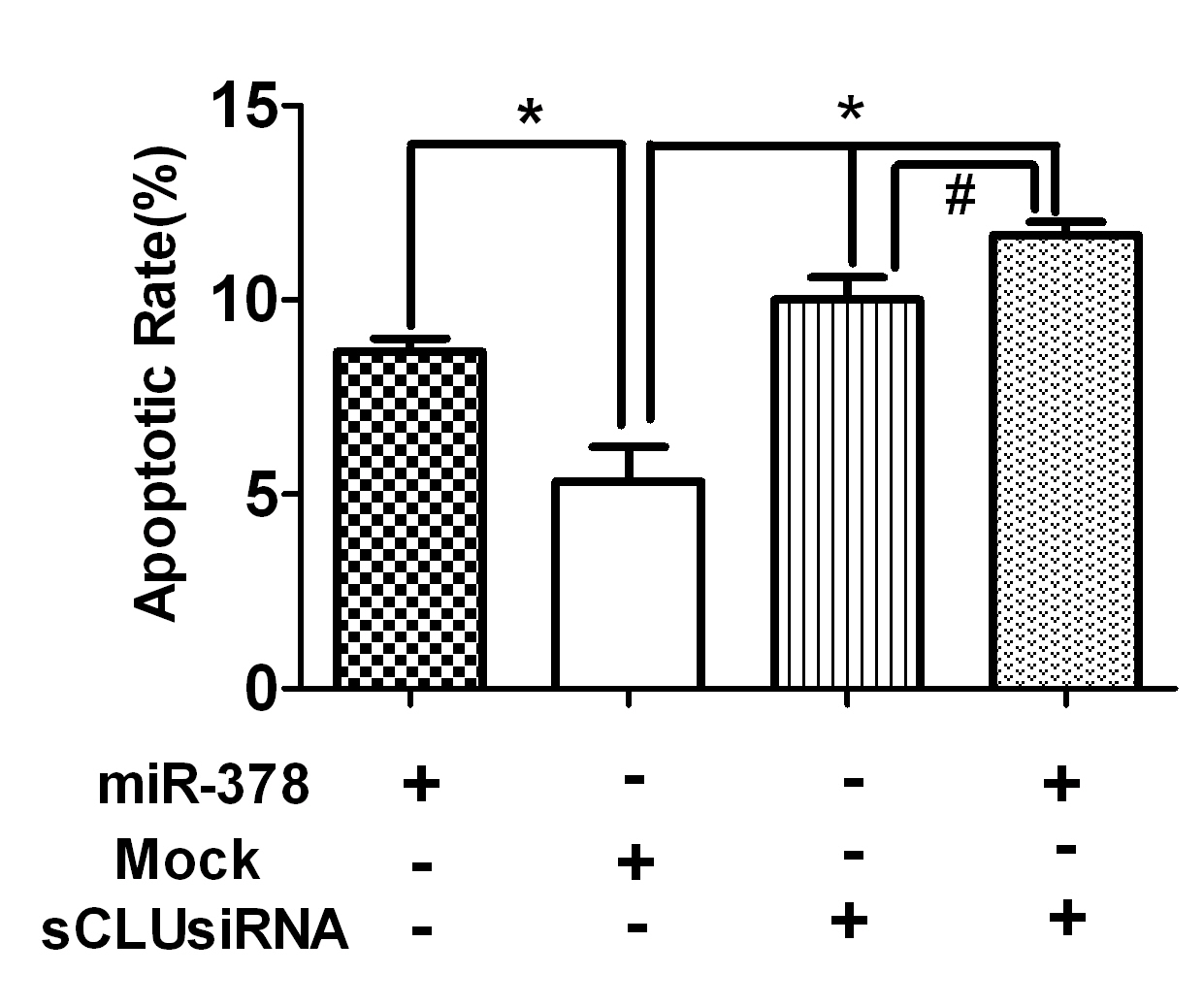


**Supplementary Figure 3. miR-378 affects cDDP-induced apoptosis in Anip973/cDDP cells.**

Hoechst staining assay showed that the apoptosis rate significantly increased in miR-378 overexpression cells compared with vector controls; miR-378 overexpression had no effect on apoptosis after sCLU knockdown. (*P<0.05, ^#^P>0.05).

**Supplementary information:**


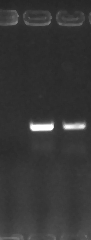

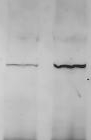

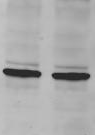

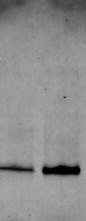


Sup-fig1c-miR-378 Sup-fig1d-sCLU Sup-fig1d-actin Sup-fig3a-sCLU


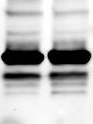

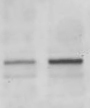

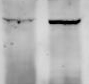

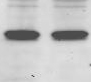


Sup-fig3a-actin Sup-fig3b-BCL-2 Sup-fig3b-pCas-3 Sup-fig3b-Cas-3


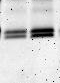

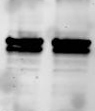

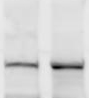

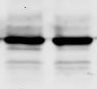


Sup-fig3b-pERK Sup-fig3b-ERK Sup-fig3b-pAKT Sup-fig3b-AKT


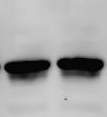

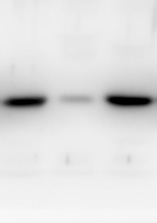

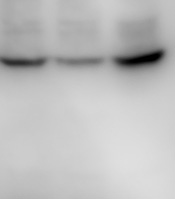

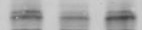


Sup-fig3b-actin Sup-fig4b-Bcl-2 Sup-fig4b-pCas Sup-fig4b-pERK


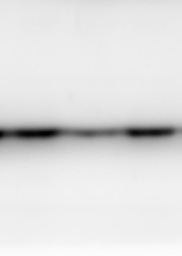

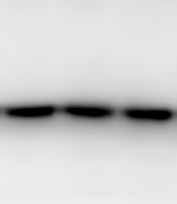

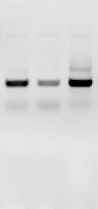


Sup-fig4b-pAKT Sup-fig4b-actin Sup-fig4b-sclu


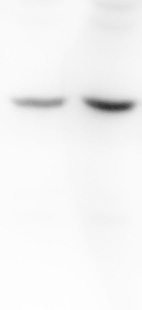

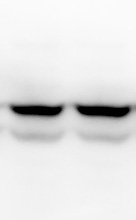


Sup-fig5b-sCLU Sup-fig5b-actin
